# Fully 3D Modeling of Electrochemical Deionization

**DOI:** 10.1021/acsomega.2c07133

**Published:** 2023-01-05

**Authors:** Johan Nordstrand, Léa Zuili, Joydeep Dutta

**Affiliations:** Functional Materials, Applied Physics Department, School of Engineering Sciences, KTH Royal Institute of Technology, AlbaNova universitetscentrum, 106 91 Stockholm, Sweden

## Abstract

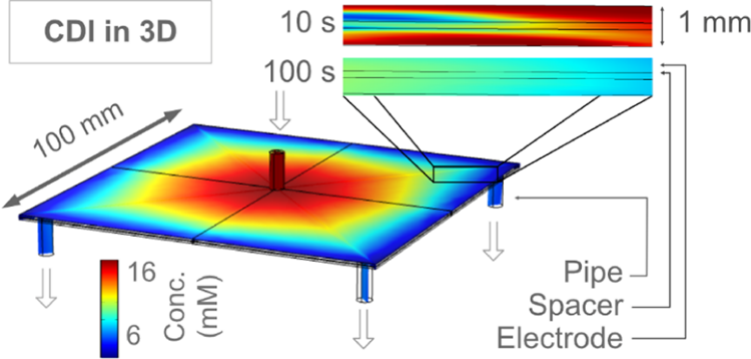

Electrochemical deionization devices are crucial for meeting global freshwater demands. One such is capacitive deionization (CDI), which is an emerging technology especially suited for brackish water desalination. In this work, we extend an electrolytic capacitor (ELC) model that exploits the similarities between CDI systems and supercapacitor/battery systems. Compared to the previous work, we introduce new implementational strategies for enhanced stability, a more detailed method of describing charge efficiency, layered integration of leakage reactions, and theory extensions to new material and operational conditions. Thanks to the stability and flexibility the approach brings, the current work can present the first fully coupled and spatiotemporal three-dimensional (3D) CDI model. We hope that this can pave the way toward generalized and full-scale modeling of CDI units under varying conditions. A 3D model can be beneficial for investigating asymmetric CDI device structures, and the work investigates a flow-through device structure with inlet and outlet pipes at the center and corners, respectively. The results show that dead (low-flow) areas can reduce desalination rates while also raising the total leakage. However, the ionic flux in this device is still enough under normal operating conditions to ensure reasonable performance. In conclusion, researchers will now have some flexibility in designing device structures that are not perfectly symmetric (real-life case), and hence we share the model files to facilitate future research with 3D modeling of these electrochemical deionization devices.

## Introduction

Across the world, over 2 billion people are experiencing water stress,^1,2^ and the rapidly expanding world populations broadly drive the need for developing effective desalination technologies.^3^ Among the available desalination technologies,^4−15^ electrochemical devices can use electrical forces to remove contaminants. One such is capacitive deionization (CDI), an emerging technique that uses supercapacitors to extract salt from water.^4,16,17^ This is promising for desalinating brackish water^18^ as well as versatile applications^19,20^ such as water disinfection,^21^ resource production,^22^ and energy harvesting.^23,24^ The structure and operation of such devices are key for sustainable water production and reusable water supplies with reduced water wastage.

A typical CDI device comprises a pair of micro-porous electrodes separated by a spacer.^25^ During the desalination operation, a voltage charges the electrodes, thereby removing salt ions from a passing water stream.^26^ Process performance^27^ in CDI depends strongly on electrode material and its microstructure,^28−41^ as well as operational^42−48^ and structural^25,49,50^ conditions of the device. Appropriate simulations of devices and structures are thus a necessary avenue for understanding, predicting, and improving device performances.

In CDI, a detailed interplay between desalination adsorption rate and migration of ions determines the efficiency of ion removal.^29,51^ Thus, while some modeling work is zero-dimensional (0D),^52−56^ others use one-dimensional (1D) spatial resolution across the thickness of a CDI device,^28,38,57−62^ 1D along the length, partial two-dimensional (2D),^63^ or full 2D.^51,64^ Some recent work even introduced simplified three-dimensional (3D) simulations for CDI^65^ and coupled 3D steady-state simulations of flow-electrode CDI.^66^ Such spatial simulations could identify concentration shocks,^51^ local dead zones in the device,^51^ flux distributions in new structures,^66^ etc. However, the complex interconnected processes in CDI can make modeling unsteady,^51^ which limits the detail and applicability of coupled spatiotemporal simulations in higher dimensions.

Recently, we introduced an electrolytic capacitor (ELC) model for CDI.^67^ The model used a new approach based on merging battery-style theory with key concepts in CDI. This allowed it to be adaptable to a variety of tasks, and that work mainly investigated leakages and multi-ion distributions in 2D. Here, we develop theories and implementations that make it possible to bring the model to the 3D setting by leveraging this new style of modeling to overcome the classic issues with simulating CDI in higher dimensions. Compared to classic models,^51,64^ the novelty of this model is that it has a decoupled framework that enhances simulation robustness so that 3D simulations can be performed. It also includes leakage descriptions and multi-ion solutions in the core theory framework. Compared to the earlier ELC,^67^ this work also contains new implementational methods for the 3D setting and a more detailed description of charge efficiency at low concentrations. We further pave the way toward a truly generalized model by presenting theory for how that connects to extended material and operational conditions, such as intercalation materials.

To the best of our knowledge, this is the first fully coupled and spatiotemporal 3D model in CDI. With this model, we investigate a square asymmetric CDI design that cannot be fully resolved in fewer dimensions^49,68,69^ that has been hypothesized to have issues with dead no-flow zones.^49^ Compared to earlier works, this work thus presents the novel ability to investigate pipe connections, intercell flow modes, and other complex asymmetric structures that can become relevant in real-life device design Put together, the goal here is to develop a general framework for electrochemical deionization processes on a full 3D scale.

## Theory

This section will present the 3D-enabled ELC model, including the derived theory that enables such 3D modeling. The first subsections will present battery-style circuit modeling that makes it possible to identify and separate the contributions to the total current. The latter section will convert this to an adsorption formulation in CDI by deriving extra stable formulations of the charge efficiency that fit within the wider framework.

### Charging in 0D

A good description of charging in 0D is important for understanding what type of behavior we would like the 3D model to describe. It will also help with parameter fitting down the line.

**Figure 1:**
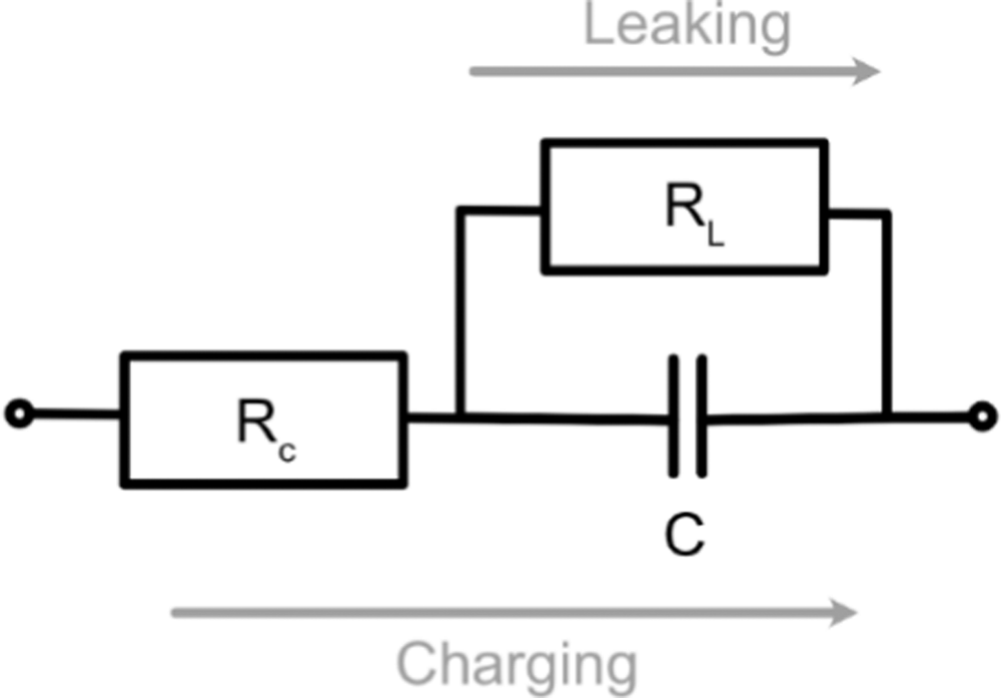
Randles circuit representing the CDI device. The total applied voltage is *V*_ext_, and the current can either contribute to charging the device (via the *C* capacitor) or leak through it (via the *R*_L_ resistance). These circuit parameters are of important consideration while refining the model to include spatial information inside the CDI devices.

The CDI unit is fundamentally a capacitor, and it desalinates water while charging. The current passing through the device can either contribute to charging or leak through the device. Also, the charging rate depends on the overall resistance in the circuit. Thus, a simplified electrical model describing the system in 0D is the Randles circuit (Figure 1),^70,71^ with a capacitive element *C*, a serial circuit resistance *R*_c_, and a leakage resistance *R*_L_.

With the charge in the capacitor being *Q*, we denote the following derivations of currents as *Q̇*, *i*_c_, and *i*_L_, respectively. Also, the external voltage is denoted as *V*_ext_. Equations 1–3 show the governing equations for such a circuit.
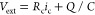
1
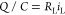
2
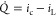
3By inserting eqs 2–3 into eq 1, we obtain a differential equation for the current (eq 4). Equation 5 shows a rearranged version of eq 4 where *t*_0_ ≡ (1/*R*_c_ + 1/*R*_L_)/*C*. While this equation holds for time-varying voltages, in classical operation conditions with a constant voltage, a neat analytic solution is obtained as shown in eq 6.
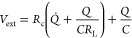
4
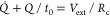
5
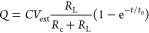
6In the last step, by inserting eq 6 back into eq 1 and simplifying it, we obtain an analytic expression of the overall current as a function of time (eq 7). A nonlinear fitting method could be used to extract *R*_c_, *R*_L_, and *C*, based on measured time-series data of the overall current from any actual measurements carried out during such operations. These known parameter values then make it possible to predict the performance of new operations.
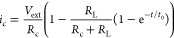
7While this 0D model provides some information about the macroscopic system properties, a model with spatial resolution would be able to provide a better peek inside the device. This could provide crucial information on the device structure and how the operation affects the efficiency of the CDI process. Therefore, in the following sections, we derive equations that provide this spatial resolution, and finally, in the results section, we investigate in 3D how the device structure can affect a CDI process.

### Electrode Current

The core idea in the ELC model is to view the contributions to the current separately, and semiseparate from the adsorption. This will help in bringing stability and adaptability.

To start, we have shown how an external voltage *V*_ext_ drives the CDI charging process at some total current *i*. Before this current reaches the electrodes, there are some potential losses due to the circuit resistance *R*_c_. Thus, eq 8 describes the potential at the electrode boundary ϕ_s,b_.
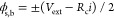
8If the electrode is highly conducting, the current passing through the electrode matrix is almost instantaneous and the entire electrode matrix experiences the same potential ϕ_s_. Otherwise, the electrode matrix conductivity σ_s_ and the electric field ∇ϕ_s_ will determine how quickly the current ***i***_***s***_ moves through the electrode (eq 9). Inside the electrode matrix, the total current distributes across the electrode volume *S* (eq 10) and passes to the electrolyte at a localized current rate *i*_v,tot_ (eq 11). While some earlier work assumed the electrodes to be highly conductive,^51^ this makes it possible to incorporate electrode resistance that is separate from the external resistance.
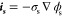
9
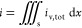
10
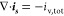
11

### Current Types

What determines the localized current rate inside the electrode matrix? Analogous to the 0D model in Figure 1, the localized current that passes from the electrode matrix to the electrolyte can be either a capacitive current that contributes to desalination or unwanted leakage currents (eq 12).
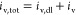
12The charging current *i*_dl_ depends on the double-layer capacitance *C*_dl_ and how quickly the difference between the electrode matrix ϕ_s_ and the electrolyte potential ϕ_l_ changes (eq 13). As the CDI device charges, the electrode potential will typically rise and reach a fixed value if the external potential is constant, while the electrolyte potential will approach zero as the device moves toward being fully charged.
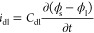
13On the other hand, the higher potential differences also drive quicker leakages. Thus, the Butler–Volmer equation (eq 14) describes how the leakage current *i*_v_ depends on the exchange-current density *i*_0_, the anodic/cathodic transfer coefficients α_a_ and α_c_, the Faraday constant *F*, the gas constant *R*, and the temperature *T*. For reasonably small voltages, a Taylor expansion of the exponentials simplifies this expression to the linearized Butler–Volmer equation (eq 15), showing that a constant leakage resistance *R*_L_ = *RT*/*i*_0_ (α_a_ + α_c_)*F* is indeed determining the leakage current of any device (eq 16).
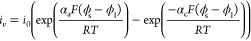
14
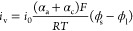
15
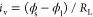
16

### Electrolyte Current

So far, we have demonstrated how the current passes through the circuit and from the electrode to the electrolyte. On the electrolyte side, charging and leakage currents generate a corresponding current ***i**_**l**_* in the electrolyte (eq 17). In the electrolyte, the flux ***N***_***i***_ of charged ionic species with valency *z*_*i*_ determines the rate of change of current (eq 18, *i* is the index for each species). This ultimately means that it is important to understand the ion flux to be able to predict the overall charging rates of the supercapacitors.
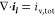
17
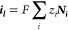
18The ionic flux arises from diffusion (the diffusion constant is *D*_*i*_ for a species with a concentration *c*_*i*_), electromigration (mobility *u*_m*,i*_), and convection (flow velocity ***u***) (eq 19). Hence, eq 20 shows the total flux which determines the total current.
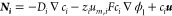
19
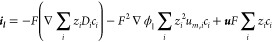
20While the total expression is quite complicated, fundamental physics allows some simplifications. The bulk electrolyte is electroneutral, making ∑_*i*_*z*_*i*_*c*_*i*_ = 0. Also, considering the diffusion coefficients to be similar, the term ∑_*i*_*z*_*i*_*D*_*i*_*c*_*i*_ would be negligibly small compared to that from migration. For mobility, we use the Nernst–Einstein relationship *u*_m*,I*_ = *D*_*i*_/*RT*. Put together, this leads to eq 21. Finally, as we primarily focus on the desalination of water, we mainly consider binary monovalent electrolytes in this study (NaCl and KCl). This means *z*_*i*_ = ±1, and *c*_–_ = *c*_+_ = *c* because of electroneutrality. Put together, the current can then be expressed as in eq 22, where σ_*l*_ is the conductivity of the electrolyte.
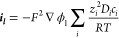
21
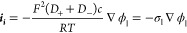
22

### Adsorption Rate

This section shows a derivation of a more detailed description of the adsorption rate than has been derived in earlier versions of the ELC model.

A stability issue with classic modeling approaches is that they used large sets of interconnected PDEs and quasi-equilibrium conditions to describe adsorption. Therefore, a key part of the new model is to reformulate the problem of adsorption into a differential form that can be solved directly. However, the new formulation should also retain a detailed description of the adsorption behavior over a wide range of operational conditions.

The double-layer current *i*_v,dl_ corresponds to ions leaving the solution to fill the electric double layers at the electrode surface at a rate *R*_*i*_ (eq 23). This is the differential form of adsorption. Ideally, such a current would correspond to *i*_v,dl_/*F* ions. Every salt molecule in the monovalent binary mixture has two charges (*n* = 2), so the current equivalently corresponds ideally to *i*_v,dl_/2*F* salt molecules leaving the solution.
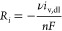
23However, real charging is not ideal, and the difference corresponds to a stoichiometric coefficient of adsorption ν that is less than unity. One reason for such nonideal charge efficiency to occur is due to co-ion expulsion. During charging, the electrode creates a charge imbalance mainly by adsorbing new ions. This would correspond to an ideal *ν* = 1. However, the electrode could also create a charge imbalance by expelling ions of the opposite charge sign that were present on the electrode from the start (such as due to passive adsorption to neutralize charged groups or passive presence due to the concentration in the liquid). When the electrode expels ions instead of adsorption new ions, this means the net adsorption is less than ideal.

The brief derivation is shown below. Because of unideal charge efficiency, we recognize that the concentration of charges in the double layers *q* (unit mol) is separate from the concentration of salt *w* (unit mol).^51^ Even in net uncharged electrodes (*q* = 0) there will typically still be some salt content *w*_0_ corresponding to an equal concentration of anions and cations. To be specific, *w*_0_ = *c* exp (μ̅_att_), so *w*_0_ is proportional to the concentration *c* and a constant fitting parameter exp (μ̅_att_). Thus, when *q* increases due to charging, the part of *w*_0_ corresponding to co-ions will escape to the solution and thus reduce the net adsorption onto the electric double layers. Quantitatively, the balance between *q* and *w* depends on the Donnan potential Δϕ̅_D_ as in eqs 24 and 25 for typical monovalent electrolytes.
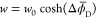
24
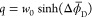
25Knowing this, *w* can be expressed as a function of *q* (eq 26) which leads to an expression for the net charge adsorption (eq 27). Experiments typically deduce the total charge (∑, unit C) and adsorption (Γ, unit mol) at equilibrium, and by multiplying with the electrode volume *V*_e_ (Γ_0_ = *w*_0_*V*_e_) we can extract the value of *w*_0_ from experimental data (eq 28).

Recall that one of the classic issues with stability is the large interconnected PDE set.^64^ Some works have suggested linear decoupled formulation to enhance stability.^53−55^ In contrast, eq 28 presents the same nonlinear results as a fully coupled model, gaining full accuracy while keeping stability.
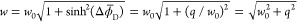
26
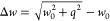
27
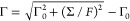
28Returning to the estimation of charging efficiency, this result now renders it possible to deduce *ν*; that is, how much the net charge concentration changes when the concentration of charges available for adsorption on the electrodes increases due to the rising potential (eq 29, taking the *q* derivative from eq 26). Because *q* is the localized charge concentration, it simply follows from the localized current as in eq 30. With this formulation, we have thus gained a differential-form description of the adsorption that combines decoupled stability with nonlinear accuracy.
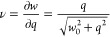
29
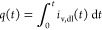
30

### Ionic Transport

So far, we have derived the generated current in CDI due to the adsorption of ions. In this section, we translate the ion adsorption to a net desalination output of the water where salt ions contribute to forming the aqueous electrolyte.

Equation 31 shows the mass transport equations for the salt. The transport mainly constitutes diffusion (salt diffusion *D*) and convection (flow ***u***), while the adsorption *R* removes ions from the solution. In the porous regions, the porosity reduces the available space for the salt and reduces the effective diffusion (eqs 32 and 33, Bruggeman equation). Finally, we used the Brinkman equations to simulate the water flow inside the device (ref (72)).
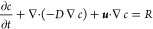
31
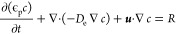
32
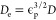
33

### Advanced Mechanisms

The previous section made some simplifications regarding the electrolyte composition, the electrode composition, and the operation.

First, we have assumed a binary solution of monovalent ions. One reason for this is to get a straightforward expression for the conductivity, as shown in eq 22. However, the core expression in eq 21 is not dependent on these assumptions and works also for multispecies mixtures that include ions with higher valences. As an additional point, the conductivity formula is decoupled from the rest of the model in the sense that it could be replaced with a different formulation if necessary. So, the formulation could be replaced with a more detailed theory or even tabulated conductivity trends for different ionic mixtures. Still, we find that the present formulation is sufficient under the conditions investigated here, especially since a large part of the resistance is in the contacts. Put together, divalent ions are covered by the theory although not modeled explicitly.

The multispecies electrolyte is more complex and also requires that ions are described separately with regard to transport and adsorption. The concentration of each concentration on the electrode can be fully described as *c*_*i*_ = *c*_*i*,0_ exp(μ̅_att,*i*_)exp(*z*_*i*_Δϕ̅_D_). The total adsorption is then a sum of all species concentrations, while the net charge is the sum of all concentrations weighted by the valency of each species. The fitting parameters μ̅_att,*i*_ are separate for each species and could be used to determine the time-dependent adsorption of each species. An example of this method implemented can be seen in ref (67). Practically, we can see that simulations of multispecies solutions introduce complex interactions that reduce the typical simulation accuracy.

Second, the discussion on the material behavior assumed double-layer adsorption on standard electrodes. However, researchers use other materials as well, such as intercalation materials, membranes (MCDI),^73^ or flow-electrode (FCDI).^74^ Intercalation materials are different in that they adsorb via reactions and diffusion into the intercalation particles can be slow. This situation can be modeled via an extra 1D dimension corresponding to the thickness of the intercalation material. The Butler–Volmer equation (eq 34) thus determines how quickly the particles charge instead of eq 13. Here, η is the overpotential (eq 35). Also, the potential drop across the electrode is not governed by the standard capacitor equation (eq 13) but rather via the Nernst equation (eq 36). Here, is a constant reference potential *R* is the gas constant, *T* is temperature, *n* is the number of participating electrons, is the concentration of reduced species, and is the concentration of oxidized species. Inside the particles, there is inward transport from the surface, following Fick’s law (eq 37). Here, *c*_p_ is the concentration inside the intercalation material and *D*_p_ is the diffusion coefficient in the intercalation material. An example of a study that models the inside of the electrode is ref (75).
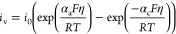
34
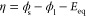
35
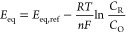
36
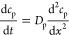
37MCDI and FCDI are not explicitly included here, although they are interesting targets for an extension. It should be noted that the difference between MCDI and CDI is largely implicit in the charge efficiency parameter. Some previous work has been able to apply models without explicit membrane description to MCDI devices with good results.^54^

Finally, the initial formulation in eq 1 used an external voltage to describe the total current in the system. This, however, does not exclude the use of constant-current (CC) charging. Using FEM software such as COMSOL, a constant-current voltage *V*_CC_ can be set implicitly [*V*_CC_*|i* = *i*_CC_] by requiring that the current *i* is the same as the desired constant-current level *i*_CC_. As an example, consider ref (76).

The point of this section is the explain the context around the model to see how it can be extended to nearby applications. Because this study mainly focuses on 3D modeling, we will mainly examine the simpler case of monovalent binary electrolytes in the Results section.

### Boundary Conditions

COMSOL automatically handles the internal boundary conditions in the model and ensures the conservation of mass and current. The outer boundaries are “walls”, where no external mass/current flux occurs and where the water flow rate is zero. To simplify the computations, we exploited symmetries to model only part of the device. The symmetry boundaries are also based on no-flux conditions.

The inlet is described with a constant concentration and a constant flow velocity. The outlet is described with a zero concentration gradient because the concentration is constant after leaving the device. Also, the pressure is set to zero so that the velocity is calculated automatically based on the inlet flow velocity.

### Parameter Fitting

The key parameters that govern the supercapacitor charging process are *R*_c_, *R*_L_, and *C*. We extracted these using time-series data for the total current upon constant voltage charging, eq 7, and MATLAB’s *lsqnonlin* solver function (nonlinear least-squares solver). Similarly, *w*_0_ governs the charge efficiency, and the *lsqnonlin* function would then extract the parameter based on the relationship between the equilibrium charge and voltage in the same experiment (eq 28). Other parameters such as porosity and diffusivity are taken from experimental measurements and tabulated values.

## Methods

### Implementation in COMSOL

COMSOL has extensive support for simulating supercapacitors, batteries,^77^ and porous flows.^72^ However, there are key differences between CDI and a classical battery system. Mainly, the ion concentrations are lower, the charging process is predominantly onto electric double layers, and the charge efficiency is important when evaluating performance. Hence, a key idea in this work is to streamline CDI modeling by leveraging the existing support in COMSOL before extending the model to solve other critical issues in CDI operations. In this model, “Brinkman equations” are used to simulate the background water flow and the “Transport of Diluted Species” interface to simulate ionic transport. The battery-based “secondary current distribution” solves both the current and potential distribution during the charging process. To address the charging efficiency calculations, a separate “domain ODE and DAEs” (ordinary and algebraic differential equations) interface was implemented in eq 29, thus effectively estimating the stoichiometric *ν* parameter in eq 23.

### Stabilization Techniques in COMSOL

A full 3D model can be both computationally taxing and unstable. To ensure smooth performance we implemented the following:

- Integrated the derived equations in COMSOL’s existing interfaces wherever possible to leverage built-in stabilization schemes.
- Started the computations at equilibrium.
- Smoothly and gradually increased the voltage at the beginning.
- Solver configurations were set to “Automatic Newton”.
- Anisotropic meshing was done to resolve issues with the thin electrode-spacer layers.

As a clarification to the last item, we can clarify that a challenge with the 3D system is the geometrical scales in different directions. The charging dynamics mainly depend on the resolution along with the thickness, meaning a lot of mesh points are needed. Fewer are required across the length and width. Thus, we chose an anisotropic mesh with a denser resolution in thickness.

### Experiments from the Literature

To evaluate how well this new model performs, we benchmarked it with the seminal work by Hemmatifar et al. which documented the first fully two-dimensional CDI model to simulate spatiotemporal dynamics in a flow-between CDI device.^51^

### Experimental Results from Our Group

**Figure 2:**
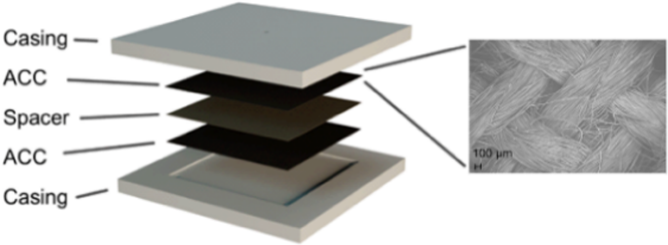
Structure of the CDI device. The plexiglass casing housed ACC electrodes, a filter-paper spacer, and graphite current collectors (not shown). Water enters through a tiny hole at the top and exits from four tiny holes at the bottom corners. Because the electrodes are more permeable than the spacer, the water flow will be much higher in the electrode than in the spacer. Enlargement: A scanning electron microscopy (SEM) image of ACC. Unlike a flow-between mode where diffusion is the main transport mechanism in the electrodes, in the flow-through mode, salty water flows through the highly porous electrodes.

The primary experiments in this work used a square flow-through CDI device as described elsewhere (Figure 2).^49^ This comprised two pieces of activated carbon cloth separated by a filter-paper spacer. Together with current collectors, the entire assembly was housed in a plexiglass casing. The casing was constructed so that the water enters from a small hole at the top and exits through four holes in the corners at the bottom, wherein it is expected that water flows through the entirety of the electrodes on the way from the inlet to the outlet.

Specifically, the electrodes used commercially available activated carbon cloth (Chemviron, Zorflex FM10) of 10 cm × 10 cm and a thickness of 0.5 mm. The as-received electrode materials were cleaned in 2 M nitric acid for 24 h, followed by thorough rinsing with deionized water and drying at 150 °C in an oven before its use.

Unless otherwise specified, the experiments used 1.2 V for charging, 0 V for discharging, 10 mL/min flow rate, and 51 mM (3000 PPM NaCl) inlet ion concentration. The conductivity of deionized water was measured at the exit of the device using an online EPU357 eDAQ conductivity meter while a Keithley 2110 multimeter was used for determining the current and voltage.

The operational conditions were chosen such that the main focus of the study is adsorption behavior rather than leakage behavior. More detailed investigations for higher voltages can be found in ref (67). However, the theory used here to describe leakages at lower voltages than the water-dissociation voltage is consistent with the mechanisms at higher voltages too (Butler–Volmer equations). The concentrations are chosen to represent a typical concentration range.

## Results

In this section, we introduce the three-dimensional CDI model for flow-between and flow-through systems. Primarily, we investigated the coupled flow and adsorption dynamics in a square CDI device.

### Validating the Model

**Figure 3:**
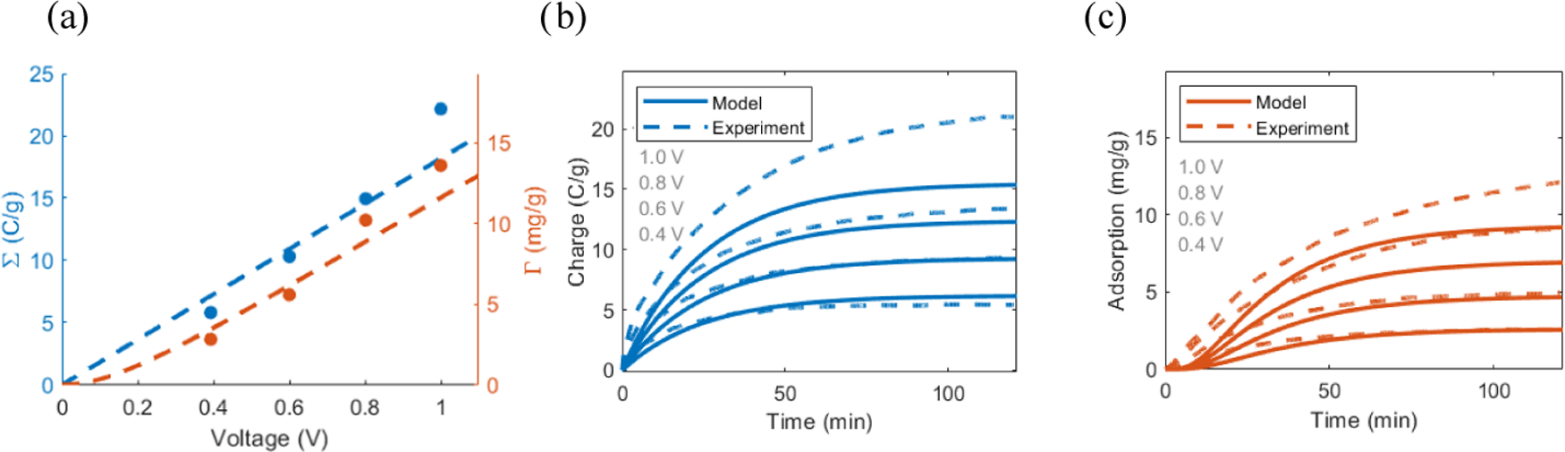
Experimental data and simulations testing for the model performance with various applied voltages for the flow-between structure (data are extracted from ref (51) while the simulations and graphics are new). (a) Equilibrium specific charge storage and specific adsorption. Dots correspond to the experimental data, while the lines are the model fits, based on eq 13 (constant capacitance) and eq 28 (charge efficiency). (b) Time-varying cumulative specific charge for the model and experiment. The model fit used the 0.6 V data set to extract the resistance, capacitance, and charge efficiency (*w*_0_) parameter. Based on that fit, predictions of the performance for the other voltages were simulated. (c) Time-varying specific adsorption for the model and experiment.

To validate the basic model setup and show that it works for a variety of cell structures, we first tested it with data obtained from a flow-between CDI cell (data were extracted from ref (51)). The equilibrium data shows that the model is satisfactory for describing variations in the applied voltage (Figure 3a). The constant capacitance parameter suggests that the stored charge should increase linearly with the voltage (eq 13), while eq 29 predicts that the adsorption increases linearly only at higher voltages when all co-ions have been expelled already. Notably, this work extends an earlier version of the ELC model by including nonlinear adsorption effects at low voltages.

Figure 3b,c further shows the model performance with a data set collected at 0.6 V to predict the performance at a range of voltages. The model works quite well for time-varying changes in charge and thus ion adsorption, Crucially, the timescales of charging and adsorption are accurate, showing the model’s tractability. Here, the charging mainly follows an RC timescale but at ion-starved conditions (when most of the ions in the solution have been removed), a high solution resistance leads to some abnormalities. The ion content reduced in the output water lags behind the charging, which is partly because it takes time to transport the ions inside the device and partly because unideal charge efficiency is mainly an issue at the beginning of each desalination cycle.

A deeper examination suggests that the significant errors in the time-dependent simulation mirror those in the equilibrium data. These errors mainly arise from the nonlinear increase in equilibrium charge stored at higher voltages (nonconstant capacitance, see ref (51) for comparison). In some works, this is addressed by introducing an empirical square dependence between the capacitance and the charging state.^64^ Looking at works without this correction factor, the dynamic Langmuir (DL) model is more numerically accurate because that model does not require that the adsorption zero-intercept is at zero volts.^67^ Some of the inherent physical square dependence is thus implicit in the model. Looking at earlier works on the mD model, the average error is approximately the same.^51^ However, the model presented here is more accurate for lower voltages and less accurate for higher voltages. This is mainly a numerical artifact from using the 0.6 V measurement to predict the entire data range, as opposed to fitting all data simultaneously. In summary, the physical errors can mainly be attributed to nonlinear capacitance, and the variations compared to similar models are mainly due to the numerical approach.

### Flow-Through Simulation in 2D

**Figure 4:**
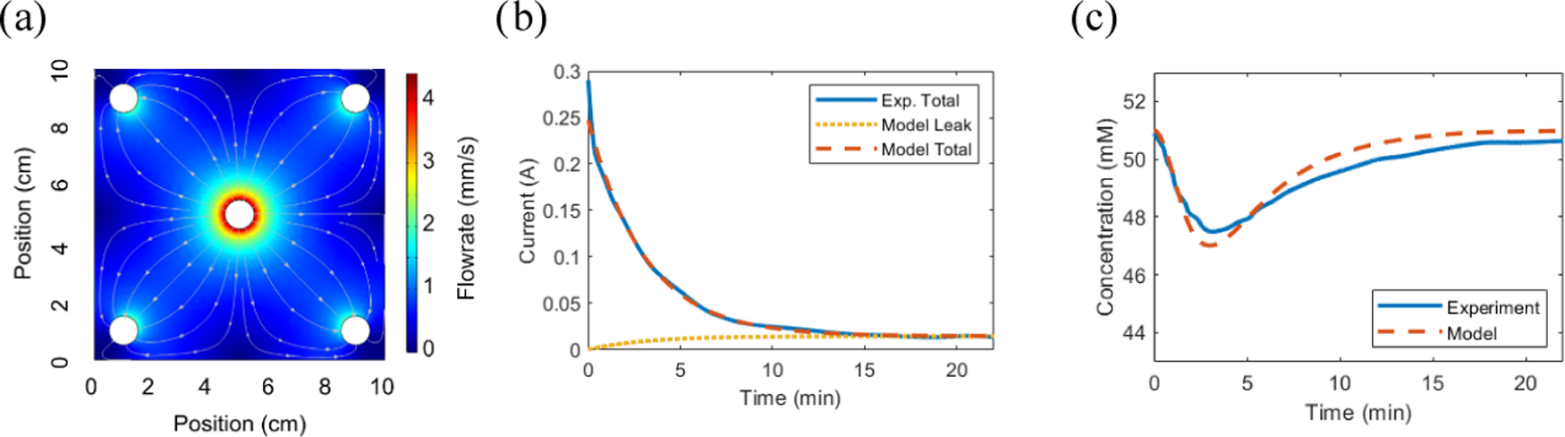
Performance of the asymmetric flat CDI structure from Figure 3. (a) Simulated flow pattern inside the device, from a top-view perspective. Because the 2D model does not resolve the individual electrode/spacer layers, we used the most permeable material to represent the effective permeability. The color shows the flow velocity, while the streamlines show the flow direction. The total flow rate is 10 mL/min. (b) Total current through the device and a model fit of a Randles circuit (Figure 1) that accounts for both the charging current and leakage current. The current that is presented here is the total current response for an operation with a 1.2 V charging voltage and 51 mM inlet ion concentration. (c) Comparison of the experimental effluent concentration to the predicted effluent concentration when the model assumes that the current uniformly distributes across the width and length of the device.

Following the validation of the model, we will now primarily focus on the flow-through asymmetric CDI structure (square) to understand how the transport pathways affect desalination performance. Because the inlet is in the center of the CDI structure and the outlets are in the extreme four corners, a top-view 2D model demonstrates that most of the water flows along the diagonals (Figure 4a). Crucially, there are thus no-flow dead zones behind the outlet pipes and at the middle of the edges that could potentially hamper the ion transport during the process.

Because the flow inside the porous materials is pressure driven, the flow distribution is the same independent of the inlet velocity. Put differently, the flow velocity normalized by the inlet flow velocity is constant. This means higher inlet velocities will lead to better ion distribution to the dead zones. However, the throughput there is always lower than at the diagonals, so they should always have a smaller effect on the relative desalination output.

Despite the complex flow pathways, the experimental current data agrees perfectly with a fitting of the 0D Randles circuit (Figure 4b). As expected, capacitance, series resistance, and leakage resistance are the key parameters affecting the current response also for the square structure. Previous research has shown that contact resistance is the largest source of resistance,^78^ so unless the electrodes are locally ion-starved, the charging should be in the RC timescale uniformly distributed across the width and length of the device. In this case, the moderately low voltage of 1.2 V leads to modest leakages even at equilibrium. While high-leakage cases are not the focus here, the model structure would be the same for higher voltages. Still, if the leakages are predicted over a large voltage difference, the linearized Butler–Volmer equation in eq 16 should be exchanged for the nonlinear version.^71^

Using a fairly high inlet concentration of 51 mM NaCl (3000 parts per million), we thus investigated the transport presuming the current to be uniform across the electrodes. Knowing the charge efficiency, this presumption leads to an adsorption rate that is also uniform (eq 13). Here, the predicted effluent concentration agrees well with the experimental data (Figure 4c), so this approach seems reasonable for investigating the general transport principles of the device.

### Flux Pathways

**Figure 5:**
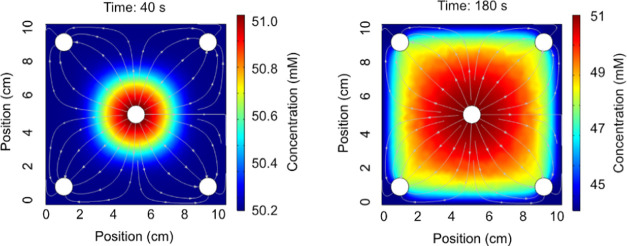
Ion concentration inside the square CDI structure during the desalination operation. The simulation used the model fit and experimental conditions from Figure 4. Here, the color scale shows the concentration while the streamlines show the directions of ionic flux. (a) Concentration and flux at 40 s after the desalination process began. (b) Concentration and flux 180 s after the desalination process began.

The spatially resolved calculations show that the beginning of the desalination process is characterized by a concentration wave front (Figure 5a). This means the current uniformly removes ions while the new ions only arrive with the inlet water. Thus, the transport rate limits which areas the replenishment water has reached yet. After a longer time, however, the inlet water reaches most of the electrode area (Figure 5b, see Supporting Information Figure S1 for constant-current mode). The time it takes will typically depend on the flow rate. In the investigated operation, it would take around a minute to replace the water in the device (∼10 mL volume and 10 mL/min flow rate). The no-flow dead zones in the corners would potentially cause concentration-starved areas where the current expends energy on removing salt ions but the cleaned water never exits the cell, thus wasting energy. This suggests that it is important to put the outlet pipes at the very edges of the corners in this type of design.

However, even though the regions at the middle of the edges also had low flow rates, the simulation indicates the transport is good enough that these electrode areas still reasonably participate in the overall desalination process. While previous work has discussed the importance of symmetry in device design, these results mean that the square design shows adequate transport throughput when the concentration is not starved.

### Asymmetric Structure in Full 3D

A model in 3D relaxes the assumption that the flow and concentration should be similar across the thickness, width, or length of the device. There are three key differences between the 3D and 2D versions of the square structure. First, the localized current depends on both the external circuit resistance, the electrode resistance, and the concentration-dependent resistance in the liquid. This means it can vary across the width and length of the electrodes. Second, the water-flow permeability is different between the electrodes and spacer, meaning there will be convective transport of ions in only the most permeable material and mainly diffusive transport from the most permeable to the least permeable region. Thirdly, potential gradients drive the localized capacitive adsorption, which means the adsorption rate will vary across the thickness of the electrodes.

**Figure 6:**
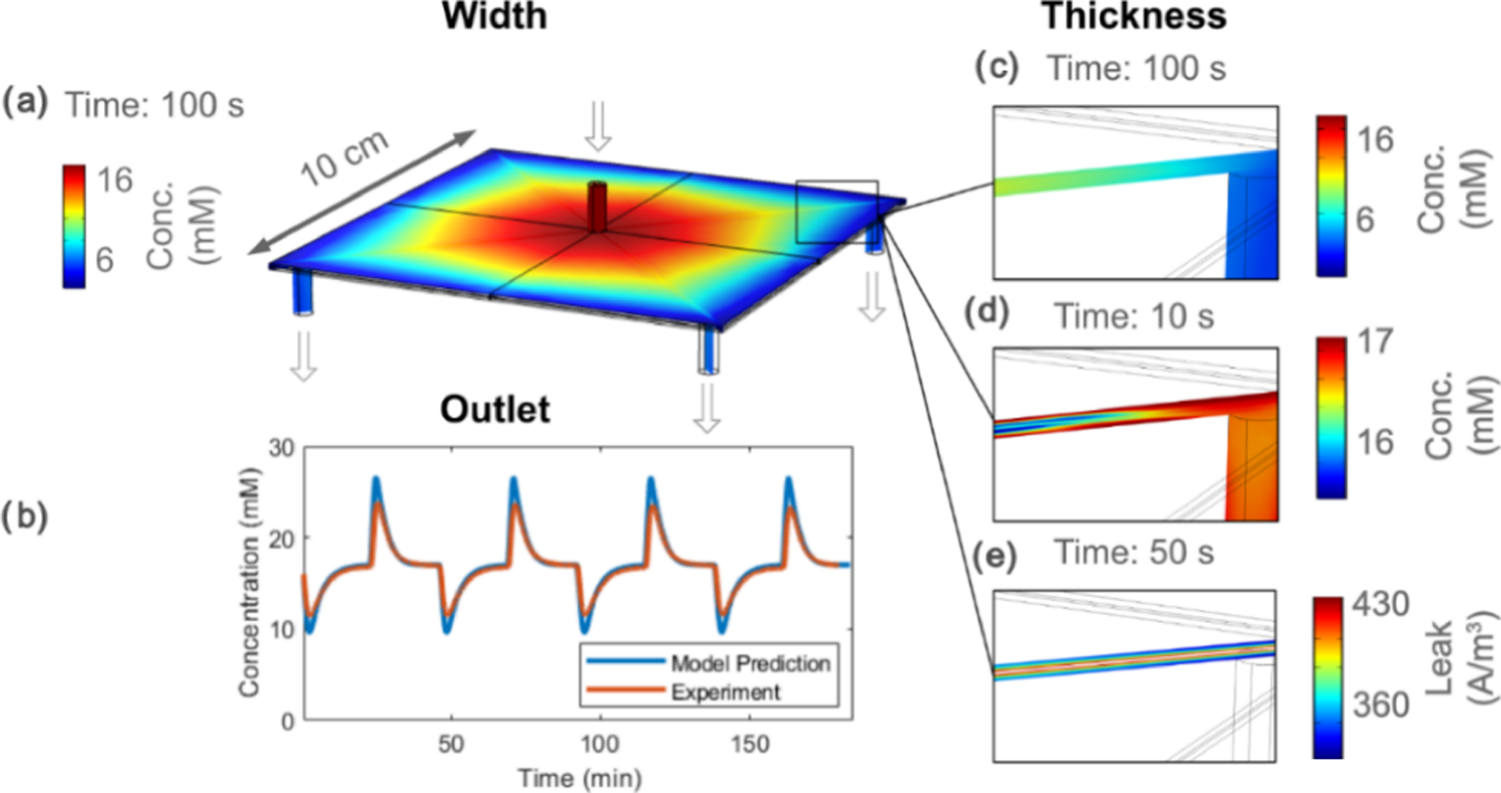
Experimental data fitted to the model for a square CDI structure. Here, the model predicted the performance at 17 mM inlet concentration based on the previous fit at 51 mM. The flow rate was 10 mL/min, and the applied voltage was 1.2 V. (a) Internal concentration inside the electrode along the width and length of the device. (b) Effluent concentration over multiple cycles of operation. (c) Cross-section coloring showing the ion concentration inside the CDI structure throughout the thickness, after 100 s of desalination. Note that the structure comprises a spacer wedged between two electrodes, and the cross section follows the diagonal path from the inlet pipe to the outlet. (d) Internal concentration after 10 s of desalination. (e) Leakage depending on the spatial location, at 50 s after the desalination process started.

Now investigating a lower NaCl concentration of 17 mM (1000 PPM), the results show a variety of key transport principles in the square CDI device. The transport and flux are similar in the 3D and 2D models across the width and length of the device (Figure 6a). A lot of ionic flux travels the shortest route between the inlet and outlet but there is also flux passing radially outward from the inlet and passing the edges before arriving at the outlet. While the model prediction somewhat overestimates the throughput rate, the predicted performance at 17 mM agrees well with experimental data (Figure 6b). The difference here could be attributed to the description of the capacitance across varying concentrations (for instance, ref (43) shows that concentration effects could be difficult to get exactly right without advanced nonlinear descriptions). However, the key finding here is that the 3D model also finds that the throughput is adequate to achieve good desalination performance because there are no major locally ion-starved regions. All water-flow pathways eventually lead from the inlet to the outlet and, like in Figure 5b. Longer 3D simulations show that the concentration near the edges keeps a similar concentration to the outlet water.

Unlike the 2D model, the fully three-dimensional model can resolve variations across the width, length, and thickness of devices. Thus, Figure 6c–e shows the performance throughout the thickness of the electrode-spacer layers. The electrode here is more permeable than the spacer which means the water flow along the length primarily goes through the electrode. However, diffusive transport along the thickness evens out the concentration (Figure 6c). On the other hand, there are differences when the desalination time is too short for diffusion (Figure 6d). At the very beginning of the desalination phase, ion adsorption starts at the electrode-spacer interface. This creates an initial variation in concentration difference throughout the device thickness.

An interesting point to note is that the parts of the electrode that are more charged also leak more. Figure 6e shows the situation at the beginning of the desalination process where charging starts at the electrode-spacer interface. Interestingly, the potential distribution determines the charging direction, so the charging direction should be the same in both flow-between and flow-through modes. The leakage strongly follows the charging and is predominant in the parts of the electrode closer to the spacer. Across the width and length of the device, the leakage situation also follows the charging.

Another perspective on the leakages comes from the resistance parts. There can be resistance in the outer contact, the electrode matrix, the solution, etc. Previous studies have argued that contacts typically have the highest contribution.^78^ Because new ions enter from the center to compensate for the removed ions, the charging and subsequent leakages are more predominant in the central regions (Supporting Information Figure S2). Another way to state this is that dead zones will raise local resistance. However, since only a part of the device has dead zones, the overall solution resistance does not change significantly. Since the solution resistance is not limiting anyway, the dead zones might not have a significant impact on the overall current (Figure 4b). Rather, the difference would be that regions with high solution conductivity are charged before the dead zones. Ultimately, this means that if the device is starved or some electrode parts are difficult to reach, the charged parts of the electrode will leak a lot while waiting for the incoming ions to diffuse out to the rest of the electrode area. This highlights that good ion-flux throughput matters not only for the desalination rate but also for reducing unwanted charge leakages.

## Discussion

As models grow bigger, computational complexity increases. Thus, two major challenges in constructing a CDI model in 3D are the computation times and the computational stability. To the best of the authors’ knowledge, this work introduces the first fully three-dimensional model for simulating desalination in CDI, which means we had to solve these stability issues. In the final implementation, the model integrates as much derived theory as possible into existing COMSOL frameworks to leverage their built-in stabilization features (such as streamline diffusion and crosswind diffusion^72^). Regarding the theory that COMSOL cannot account for, such as charge efficiency, we focused on deriving decoupled expressions that require as little extra computational complexity as possible while ensuring accurate simulations. The final model computes at around 100 min on a computer with 24 cores (a single-cluster node). To aid future research in CDI, we have made the full model and all its stabilizing features publicly available in Supporting Information Code 1. By exchanging the geometry and fitting new parameters, researchers will be able to implement the model for a variety of CDI structures.

While multiple models exist for CDI, the electrolytic capacitor (ELC) model used here was designed to leverage existing support for supercapacitors and batteries to achieve greater stability. Still, when using only a single data set for fitting, the predictions for voltages (Figure 3b,c) and concentration (Figure 6a) somewhat neglect the nonlinear (see Figure 3a) effects that changes in these parameters have on the capacitance. Thus, future studies could continue to develop the model and achieve the same tractable stability while yielding even more accurate predictions over a wider range of operational parameters.

Using the 3D model, we have shown that a square CDI structure with pipes in the corners and center can avoid ion starvation even though the design is asymmetric with respect to its flow paths. More generally, the findings suggest that CDI structures are somewhat robust to asymmetries in the design. In commercial devices, the total system cost depends on both material costs and energy costs for producing a given volume of water. Therefore, robustness is important because it allows for some leeway in the structural design; that is, some asymmetries in the design are acceptable.

## Conclusions

Electrochemical deionization is crucial for meeting global drinking-water demands, and modeling can be critical for further developing the CDI techniques. In this work, we have derived a fully three-dimensional model for such systems. This can be especially useful for investigating CDI devices that have asymmetric flow pathways from the inlet pipe to the outlet pipe.

Computation time and stability are common challenges faced in large finite-element models. Therefore, the newly derived ELC model is exploiting the similarities between CDI and standard supercapacitor/battery systems to leverage COMSOL’s strong existing support for such applications to enhance the stability and tractability of the model. However, some crucial performance metrics are outside the standard libraries, such as charge efficiency. For these, we focused on deriving decoupled expressions that would minimize the added computational complexity. The Supporting Information contains the 3D model file to aid researchers who would like to extend the 3D simulations to their systems.

This work specifically investigated a square CDI system with inlet and outlet pipes in the center and corners. Notably, the asymmetric flow pattern leads to zones along the edges where the water flow is minor. However, the results also showed that the ionic flux was enough to avoid starvation under normal operating conditions, and the device shows good performance despite the asymmetries. This finding means researchers have some flexibility when designing device structures because they do not have to be perfectly symmetric.

## Supporting Information Available

Constant-current stable internal concentration (Figure S1), leakages after 100 s of operation (Figure S2), three-dimensional COMSOL model used in this work, as well as the experimental data used for validation (Code S1) (PDF)
