# Supplementary material for: Fully 3D Modeling of Electrochemical Deionization
Source: ACS Omega. 2023 Jan 5;8(2):2607–17. doi: 10.1021/acsomega.2c07133 (PMC9850726; doi:10.1021/acsomega.2c07133)
Supplement: Supplementary file 1 — ao2c07133_si_001.pdf [file ao2c07133_si_001.pdf]

Supplementary Materials for

Fully 3D Modeling of Electrochemical Deionization

Johan Nordstrand *, Léa Zuili, and Joydeep Dutta
Functional Materials, Applied Physics Department, School of Engineering Sciences, KTH Royal Institute of Technology, AlbaNova universitetscentrum 106 91 Stockholm, Sweden
**Correspondence: johanno3@kth.se*

This PDF file includes:

Figures. S1 to S2

Other Supplementary Materials for this manuscript include the following:

Code S1

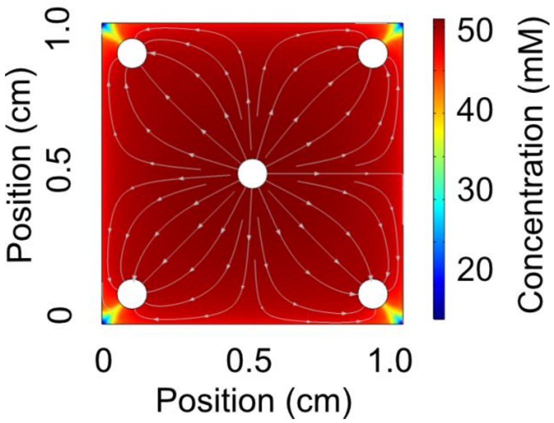

Figure S1. The ion concentration inside the CDI device during a constant-current operation at equilibrium (long-time operation). The simulation used the model fit and experimental conditions from Fig. 3. The color scale shows the concentration while the streamlines show the directions of ionic flux. Note that 'equilibrium' here means that the process has operated at a constant current long enough that ions are removed at the same rate as new ions are flowing in, and the simulations do consider the effects of saturation (high voltages after long CC operations).

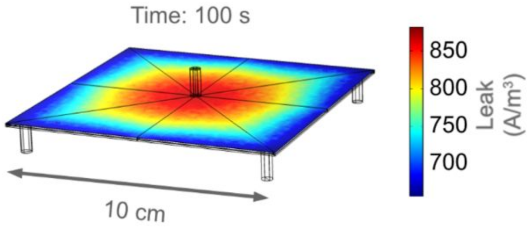

Figure S2. Leakages after 100 s of operation. The image shows the simulated results with the 3D model for the same system and operation as in Fig. 5.

Code S1. (separate file) Comsol model file for the 3D simulations. This is the model used to generate the 3D simulation results in the main manuscript. Available at:

Nordstrand, Johan (2022), "Model in 3D for Capacitive Deionization", Mendeley Data, V1, doi: 10.17632/xmjz4f2m86.1
